# Simultaneous bioreduction of tellurite and selenite by *Yarrowia lipolytica*, *Trichosporon cutaneum*, and their co-culture along with characterization of biosynthesized Te–Se nanoparticles

**DOI:** 10.1186/s12934-023-02204-0

**Published:** 2023-09-25

**Authors:** Firooz Hosseini, Maryam Hadian, Elham Lashani, Hamid Moghimi

**Affiliations:** Department of Microbiology, School of Biology, College of Science, University of Tehran, Tehran, Iran

**Keywords:** Bioremediation, Co-contamination, Kinetics, Metalloid oxyanions, Te–Se nanoparticles, Yeast

## Abstract

**Background:**

Natural and anthropogenic activities, such as weathering of rocks and industrial processes, result in the release of toxic oxyanions such as selenium (Se) and tellurium (Te) into the environment. Due to the high toxicity of these compounds, their removal from the environment is vital.

**Results:**

In this study, two yeast strains, *Yarrowia lipolytica* and *Trichosporon cutaneum*, were selected as the superior strains for the bioremediation of tellurium and selenium. The reduction analyses showed that exposure to selenite induced more detrimental effects on the strains compared to tellurite. In addition, co-reduction of pollutants displayed almost the same results in selenite reduction and more than ~ 20% higher tellurite reduction in 50 h, which shows that selenite triggered higher tellurite reduction in both strains. The selenite and tellurite kinetics of removal were consistent with the first-order model because of their inhibitory behavior. The result of several characterization experiments, such as FE-SEM (Field emission scanning electron microscopy), dynamic light scattering (DLS), Fourier-transform infrared spectroscopy (FTIR), X-ray diffractometer (XRD), and dispersive X-ray (EDX) on Te–Se nanoparticles (NPs) revealed that the separated Te–Se NPs were needle-like, spherical, and amorphous, consisted of Te–Se NPs ranging from 25 to 171 nm in size, and their surface was covered with different biomolecules.

**Conclusions:**

Remarkably, this work shows, for the first time, the simultaneous bioreduction of tellurite and selenite and the production of Te–Se NPs using yeast strains, indicating their potential in this area, which may be applied to the nanotechnology industry and environmental remediation.

**Graphical Abstract:**

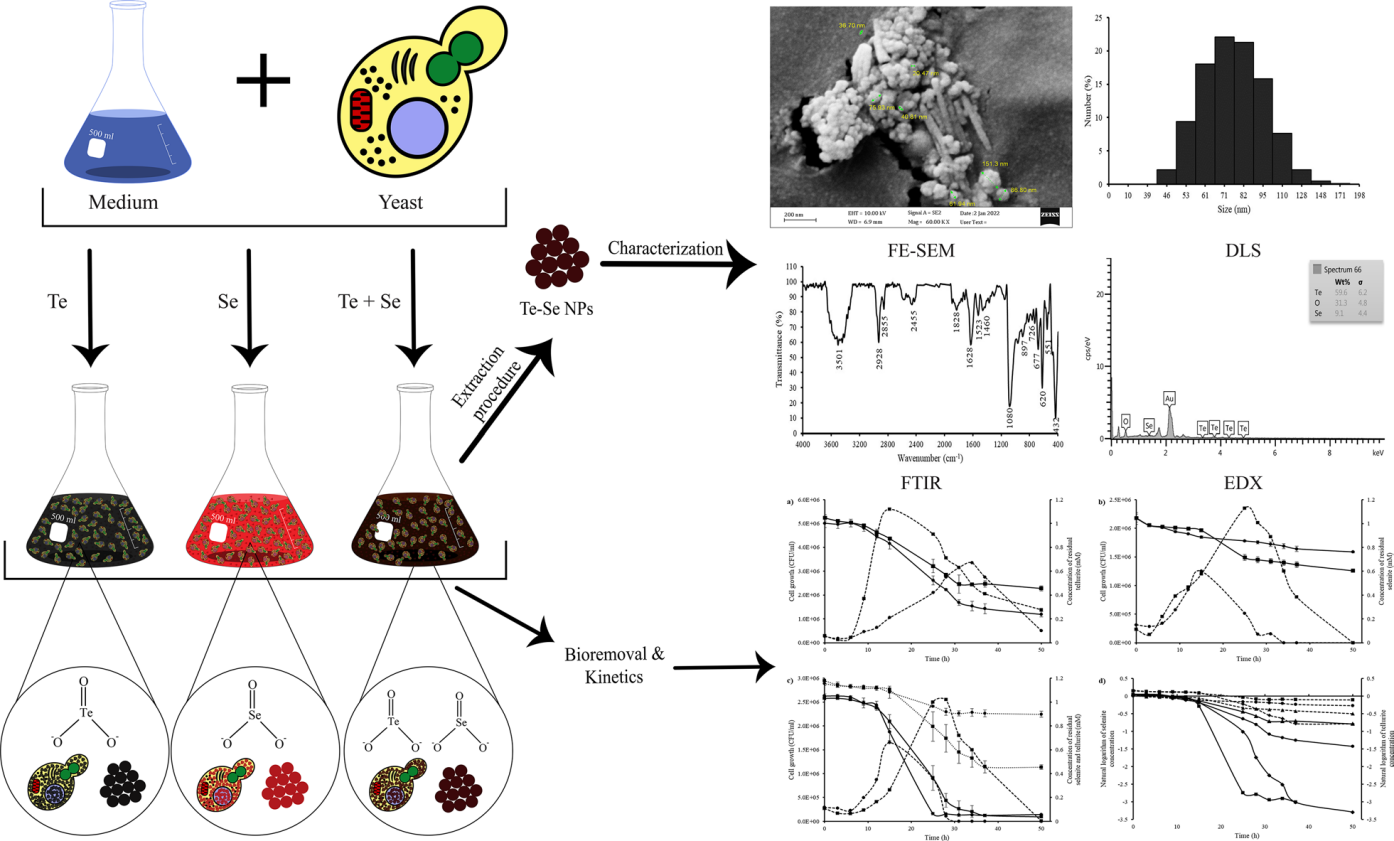

**Supplementary Information:**

The online version contains supplementary material available at 10.1186/s12934-023-02204-0.

## Highlights

- First report of co-contaminant reduction of tellurite and selenite by yeast strains.
- Higher bioremediation efficiency of co-contaminant cultures than separate cultures.
- In co-contaminant cultures, selenite triggered higher tellurite reduction for both strains.
- Bioreduction of toxic tellurite and selenite to valuable Te–Se nanoparticles.
- Characterization of biosynthesized Te–Se nanoparticles.

**Supplementary Information:**

The online version contains supplementary material available at 10.1186/s12934-023-02204-0.

## Introduction

Tellurium (Te) and selenium (Se), which are both in the 16th group of the periodic table, can exist in several states of oxidation, including + IV, –II, + VI, and 0. In nature, these elements are most frequently found in copper- and sulfur-bearing ores [1, 2], in the waste products of metal processing industries, and anode slime generated by the copper mining industry. Due to the mining and refining of ores such as nickel and copper, water and soil environments can be contaminated with tellurium and selenium [3–6].

Due to the industrial utility of tellurium and selenium, notably in technological disposals such as biotechnology, rechargeable batteries, solar panels, and biomedical devices, interest in these elements has expanded tremendously in recent years [7–10]. Concerns regarding potential human health and environmental problems have grown as a result of the rise in metalloid demand [11, 12]. Industrial wastewater, agricultural lands, and mine tailing are prone to contamination by these two elements, and their high toxicity causes environmental problems in contaminated soils and waters [13, 14].

Tellurite and selenite are the most detrimental forms of Te and Se oxyanions, which are even believed to endanger microbes at low concentrations. On the other hand, because of the low solubility and bioavailability of Te^0^ and Se^0^, they show less toxicity to microorganisms [9, 15–17]. They become exposed to the environment and frequently wind up in surface and groundwater sources as a result of natural weathering or human activities like mining [18, 19]. Other sources of tellurite and selenite include chemical and metallurgical industries, coal burning, petroleum refinery, and electro-refining processes [20–23]. Since these two oxyanions are harmful to living organisms, their removal from polluted environments is essential for pollution eradication.

Tellurite and selenite pollution is typically treated using a variety of physicochemical techniques, including cutting-edge technologies like ultrafiltration and nanofiltration [24, 25]. On the other hand, biological approaches using microorganisms can offer additional benefits for treating these wastewaters since they allow for the biorecovery and production of elemental nanoparticles (NPs) from these pollutants [9, 19, 23, 26]. A variety of microbial species can transform selenium and tellurium oxyanions through different processes, such as reduction, oxidation, demethylation, and methylation [27–29].

The promising usage of binary Te-compounds such as Te–Se composites has attracted greater interest recently. In comparison with separate Te and Se materials, these composites exhibit distinctive optical and semi-conductive properties, as well as potential usage in advanced optoelectronic and electronic devices, with improved magnetic and electrical resistance properties [30, 31].

The co-contaminant reduction of tellurite and selenite has not been investigated for yeasts, and there are only a few reports describing the co-reduction of tellurite and selenite by bacteria and fungi [18, 32–35]. For the bioremediation of tellurite and selenite, bacteria have traditionally been the preferred microorganism; however, the use of yeast in the bioremoval of these pollutants is equally promising. The natural processes of yeast cells, such as complexation, extracellular precipitation, transformation, intracellular compartmentalization, efflux systems, crystallization, adsorption onto cell walls, and pigments, allow them to rapidly adapt to metal-contaminated environments, tolerate and detoxify them [36–39]. Additionally, yeasts have the ability to employ a range of renewable carbon sources, and their biomass has been utilized to produce single cell oil and single cell protein [40–42].

Yeasts may also exploit a number of renewable carbon sources, and their biomass has been utilized to produce single cell oil and single cell protein. Therefore, this study investigates for the first time, the simultaneous reduction of tellurite and selenite by yeast strains, *Yarrowia lipolytica* and *Trichosporon cutaneum*, and the biosynthesized Te–Se NPs were extracted and characterized using energy-dispersive X-ray (EDX), Fourier-transform infrared spectroscopy (FTIR), FE-SEM, X-ray diffractometer (XRD), and dynamic light scattering (DLS).

## Material and methods

### Microorganisms and culture condition

In this study, different yeast strains were selected and screened from the Environmental Biotechnology Laboratory (EBL) collection, and their capability in tellurite and selenite reduction was evaluated qualitatively. Yeast strains were cultured in glucose yeast extract peptone (GYP) and agar medium (pH 7 ± 0.2) consisting of 20 g/L glucose, 10 g/L yeast extract, 5 g/L peptone, and 20 g/L agar supplemented with potassium tellurite and sodium selenite (48 h at 30 °C). After evaluating the reduction potentials of these yeasts in tellurite and selenite removal, the best strains were selected for further studies.

### Minimal inhibitory concentrations (MIC) and Minimum Biocidal Concentration (MBC)

For the MIC experiment, 5% (v/v) of fresh cultures (OD600 ~ 0.1) was added to 10 mL of nutrient broth (NB) medium (pH 7.2 ± 0.2), consisting of 8 g/L NB. Different concentrations of selenite and tellurite were added to the flasks of tellurite (0.5–10 mM) and selenite (0.5–20 mM). Then, the yeasts were incubated for 48 h at 30 °C (150 rpm). For the MBC test, 20 µL of each flask (above the MIC concentrations) was transferred to nutrient agar culture media and incubated at 30 °C for 48 h.

### Determining tellurite and selenite concentration

According to a method described by [43], Diethyldithiocarbamate or DDTC (10 mM) was used to determine the tellurite concentration spectrophotometrically. In this method 800 mL of tellurite cultures were combined with 800 mL of freshly made DDTC solution. Then 2400 mL of 0.5 M Tris–HCl buffer (pH = 7) was added, and the absorption was assessed at 340 nm.

The selenite concentration in the medium was evaluated spectrophotometrically by a method described by [44] with little modification. In this method, 250 µL of samples were mixed with 10 mL HCl (0.1 M), 0.5 mL NaF (0.1 M), 0.5 mL EDTA (0.1 M), and 0.5 mL disodium oxalate (0.1 M), followed by 2.5 mL 2, 3-diaminonaphthalene (0.1%) in HCl (0.1 M). After shaking the tubes, incubation was conducted at 40 °C for 40 min. Then, tubes were placed at room temperature for cooling. 6 mL cyclohexane was added to each tube and agitated vigorously for about 1 min. The upper phase was separated by centrifugation at 3000*g*, and absorbance was determined at 377 nm. The concentrations of tellurite and selenite were obtained through a calibration curve [45].

### Tellurite and selenite removal in individual and mixed cultures

All of the experiments were performed using 50 mL NB medium and incubated for 50 h at 30 °C (150 rpm). 1 mM potassium tellurite and 1 mM sodium selenite were added to each flask, followed by adding 5% (v/v) of fresh inoculum to each batch. The capability of the strains was assessed in nine different batches, including three tellurite cultures (*Y. lipolytica*, *T. cutaneum*, and co-culture), three selenite cultures (*Y. lipolytica*, *T. cutaneum*, and co-culture), and three mixed cultures of tellurite and selenite (*Y. lipolytica*, *T. cutaneum*, and co-culture).

### Kinetics and growth rate of tellurite and selenite removal

The kinetics of two yeast strains for tellurite and selenite removal and their growth rate were evaluated as individual cultures. In this experiment, six defined experimental cultures (three for each strain) containing NB medium were supplemented with 1 mM tellurite, 1 mM selenite, and the combination of tellurite and selenite, followed by adding 5% (v/v) of fresh inoculum, then incubated for 50 h at 30 °C. The growth of the yeasts was calculated by the colony-forming units (CFU) method. For investigating the growth rate and tellurite and selenite removal, about 1 ml from each culture was taken at designated times (0, 3, 6, 9, 12, 15, 25, 28, 31, 34, 37, 50 h).

Three kinetics formulas, including zero, 1st, and 2nd order were applied to obtain the described parameters. In order to determine the kinetics of tellurite and selenite removal, the following formulas were used:

1 $${\text{Zero}} - {\text{order}}:{\text{ Ct}}\, = \,{-}{\text{ K}}0{\text{t}}\, + \,{\text{C}}0{\text{ and T1}}/{2}\, = \,{\text{C}}0/{\text{2K}}0$$

2 $${\text{1storder}}:{\text{ ln Ct}}\, = \,{-}{\text{ K1t}}\, + \,{\text{lnC}}0{\text{ and T1}}/{2}\, = \,{\text{Ln2}}/{\text{K1}}$$

3 $${\text{2ndorder}}:{ 1}/{\text{Ct}}\, = \,{\text{K2t}}\, + \,{1}/{\text{C}}0{\text{ and T1}}/{2}\, = \,{1}/{\text{C}}0{\text{K2}}$$

Which Ct stands for concentration at time t, t for time, K for removal rate constant, and C0 for initial concentration [46].

### Intracellular nanoparticle biosynthesis and extraction

Both yeast strains were employed to synthesize Te–Se NPs. In this experiment, two different cultures were prepared, including combination cultures of tellurite (1 mM) and selenite (1 mM) for *Y. lipolytica* and *T. cutaneum*. In order to synthesize NPs, a combination of 1 mM tellurite and 1 mM selenite (Y. lipolytica and T. cutaneum), as well as 10% (v/v) fresh inoculums, were added to two 500 mL flasks each holding 100 mL of NB medium for 24 h at 30 °C and 150 rpm. The previously reported procedure was used to extract Te–Se NPs from the yeasts [47].

### Characterization of Te–Se NPs

Several techniques were carried out to characterize the purified Te–Se NPs, such as Fourier-transform infrared spectroscopy (FTIR), energy-dispersive X-ray (EDX), dynamic light scattering (DLS), X-ray diffractometer (XRD), and field emission scanning electron microscopy (FE-SEM) analyses. DLS technique (Zetasizer Ver. 6.01, Malvern Instruments Ltd) was employed to estimate the size distribution of purified NPs. An aliquot of purified Te–Se NPs was prepared and transferred to a cuvette to measure the size distribution of NPs at 25 °C.

FTIR analysis (Thermo, AVATAR) was used to evaluate the functional groups encapsulated in purified NPs (400–4000 cm^−1^). Purified NPs were allowed to dry and fix on aluminum foil using glutaraldehyde, coated with gold, and then examined by an FE-SEM (ZEISS Sigma 300) for size and surface characteristics equipped with an EDX operated at 10 kV. EDX was carried out on the purified NPs to assess their elemental content. The crystallinity and structure of the purified Te–Se NPs were evaluated using an X-ray diffractometer (Philips PW1730) equipment with a Cu anode (λ = 1.54056 Å) as the radiance source at a current of 30 mA and a voltage of 40 kV and with a scanning range from 10° to 80° 2θ with an angular interval of 0.05° and 1 s counting time.

### Statistical analysis

R Studio (version 3) employing R version 4.1.2 was applied for Statistical analysis of the experimental results. All the bioreduction assays were carried out in three replicates. The Levene's test and Kolmogorov–Smirnov test were used to calculate the homogeneity of variance and normality of the distribution of the investigated data, which revealed that the data showed homogenous variances with a normal distribution. The differences between the batches were compared using one-way ANOVA and then Tukey’s test. The difference was deemed significant when p < 0.05.

## Results and discussion

### Screening and selection of *Y. lipolytica* and *T. cutaneum*

**Fig. 1:**
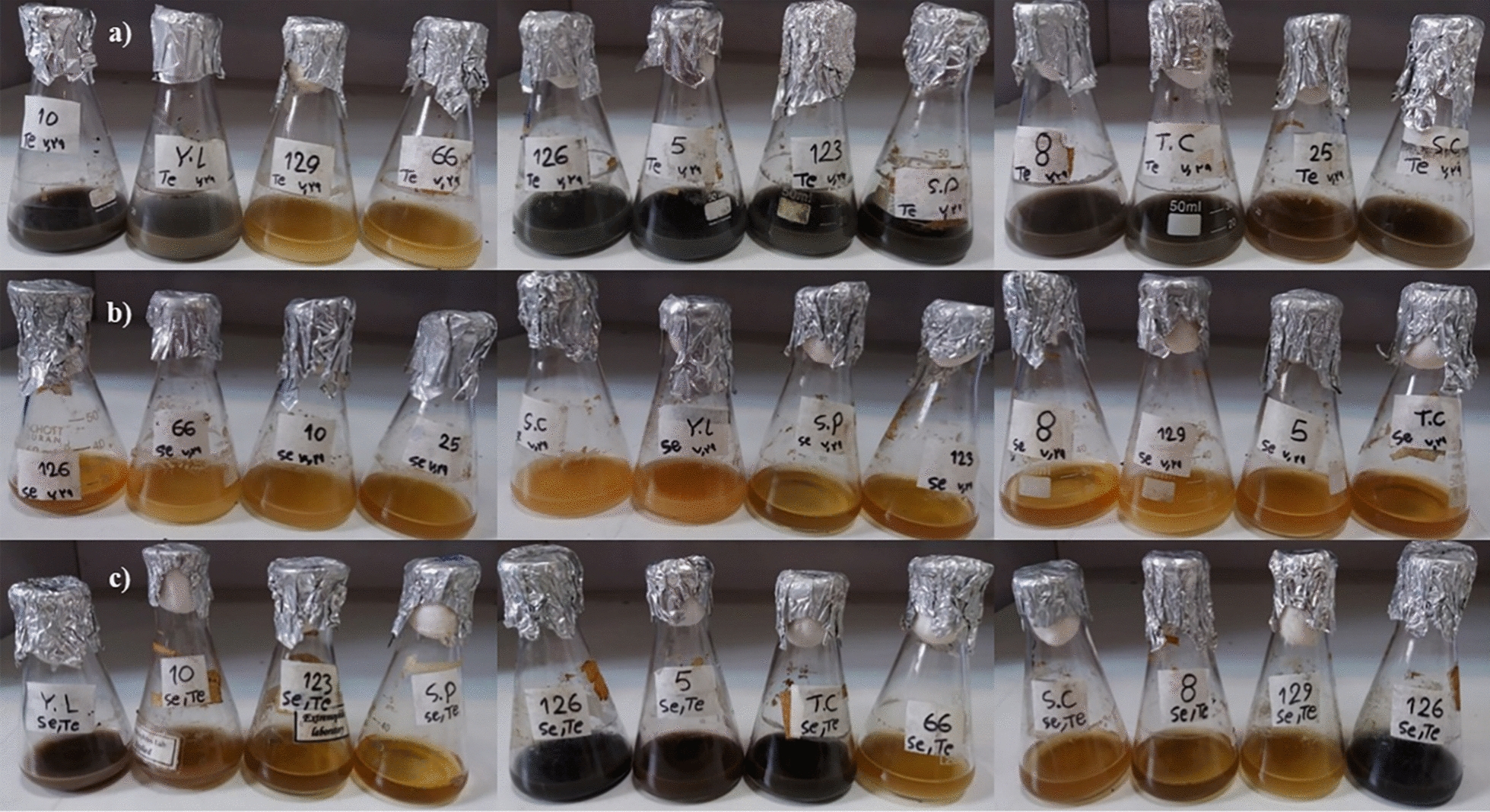
Evaluation of the capabilities of 12 different yeast strains in 1 mM tellurite (**a**), 1 mM selenite (**b**), and 0.5 mM tellurite/selenite (**c**) reduction based on color intensity after 48 h. Each flask represents a different yeast strain, which are marked by numbers and letters

The results of screening the best strains based on their tellurite and selenite reduction abilities are presented in Fig. 1, which displays the tellurite and selenite capabilities of different yeast strains based on color intensity, and the results of tellurite and selenite reduction were summarized in Additional file 1: Table S1. The results revealed that *Y. lipolytica* [48] and *T. cutaneum* [49] showed the best growth, tolerance, and reduction ability in the present of tellurite, selenite, and their mixture compared to other strains and were selected for further investigations. It is worth mentioning that tellurite, selenite, and their mixture cultures turned the media black, red, and black, respectively.

### Tellurite and selenite MICs and MBCs of *Y. lipolytica* and *T. cutaneum*

The growth of the strains was inhibited at tellurite and selenite concentrations of 2.5 mM and 12 mM for *Y. lipolytica* and 5 mM and 15 mM for *T. cutaneum*, respectively. Additionally, MBC results for *Y. lipolytica* and *T. cutaneum* were 5 mM for tellurite, 15 mM for selenite, > 5 mM for tellurite, and > 20 mM for selenite, respectively.

The tellurite and selenite tolerance of *Y. lipolytica* and *T. cutaneum* were relatively high in comparison with other studies. According to [50], MIC results of two *Saccharomyces cerevisiae* strains were more than 1.2 mM of tellurite. In a similar study, two strains of *Saccharomyces cerevisiae* were investigated, and none of them could grow in tellurite concentrations higher than 0.5 and 0.6 mM and selenite concentrations higher than 1 and 4 mM [51]. According to [52], *Candida tropicalis* and *Candida albicans* growth were completely inhibited at selenite concentrations of 29 and 58 mM. In another study, Reddy et al. reported that *Alteromonas* sp. could tolerate 7.5 mM of tellurite and 10 mM of selenite [53]. In similar studies, MIC results of a *Pseudomonas* sp*.* [54], two *Lysinibacillus* sp. [55], and *Stenotrophomonas bentonitica* [56] were as high as 150, 220, and 400 mM of selenite, respectively.

Recent studies showed the presence of Te and Se in waste products of different industries, which would result in contamination of environment. The bioremediation of these toxic compounds to less toxic elemental forms using these capable yeast strains is promising approach for treatment of metalloid oxyanions present in the industrial effluents instead of less effective and more expensive chemical and physical methods [9, 19, 23, 26].

### Tellurite and selenite removal in separate and co-contaminant cultures

Reduction assays of 1 mM tellurite and 1 mM selenite were carried out in separate cultures to evaluate the bioreduction efficiency of *Y. lipolytica* and *T. cutaneum*. Moreover, an experiment was carried out for each strain on a culture containing a mixture of 1 mM tellurite and 1 mM selenite in order to evaluate the effect of the combination of both metalloid oxyanions on the *Y. lipolytica* and *T. cutaneum*’s reduction efficiency. Additionally, in another experiment, both strains were combined to evaluate the effect of co-culture on tellurite and selenite removal in separate and co-contaminant cultures.

The results from the evaluation of tellurite, selenite, and tellurite/selenite combination reduction are presented in Table 1. It is worth noting that no abiotic reduction of tellurite and selenite were observed in control experiments (Additional file 1: Fig. S1), showing that the reduction can only be related to the yeasts’ activity. As shown in Table 1, in separate cultures, *Y. lipolytica* batches performed better in removing tellurite and showed the lowest selenite reduction efficiency within 50 h. In a similar study, *Phanerochaete* sp. reduced 40% of 10 mg/L (0.04 mM) of tellurite and 32% of 10 mg/L (0.06 mM) of selenite in 8 days [18]. In another research, *Duganella* sp. reduced 85% of 250 mg/L (1.5 mM) selenite within 15 days and 88% of 45.75 mg/L (0.2 mM) tellurite in 24 days [34]. Reddy et al. reported that *Alteromonas* sp. reduced 100% of 1 and 2 mM of selenite and 86 and 75% of 1 and 2 mM of tellurite in 48 h [53].

**Table 1:** The yield of tellurite and selenite removal in different cultures

	Separate cultures		Co-contaminant cultures	
	Tellurite reduction (%)	Selenite reduction (%)	Tellurite reduction (%)	Selenite reduction (%)
*Y. lipolytica*	76.11 ± 1.98 a	26.90 ± 4.15 a	94.60 ± 0.49 c	22.23 ± 1.46 a
*T. cutaneum*	56.45 ± 3.19 b	42.18 ± 2.86 b	96.39 ± 0.13 c	61.54 ± 0.83 c
Co-culture	74.28 ± 3.80 a	56.17 ± 3.48 c	97.01 ± 0.38 c	57.69 ± 2.37 c

Values are mean (n = 3) ± SD

Different alphabets in tellurite and selenite columns represent significance at p < 0.05 after applying post hoc Tukey’s test

In addition, while co-contaminant cultures displayed the highest tellurite reduction efficiency in *Y. lipolytica* and *T. cutaneum* batches, with 94.60 and 96.39%, within 50 h, respectively, selenite reduction in co-contaminant cultures showed no significant difference with separate cultures except in *T. cutaneum* batches. Espinosa et al. reported that in co-contamination cultures of tellurite and selenite, *Phanerochaete* sp. reduced 27% of 10 mg/L (0.04 mM) tellurite and 12% of 10 mg/L (0.06 mM) selenite in 8 days [18]. Bajaj and Winter [34] findings showed that *Duganella* sp. in the mixture of tellurite and selenite could reduce 14 mg/L (0.05 mM) of tellurite and 100 mg/L (0.6 mM) of selenite in 1.2 and 9 days, respectively. They also reported that the selenite reduction was the same in both separate and co-contaminant cultures. In another study, *Aspergillus niger* reduced 59.5% of 10 mg/L (0.06 mM) selenite and 47.2% of 10 mg/L (0.04 mM) tellurite in co-contaminant cultures within 15 days [35].

Remarkably, in the presence of both tellurite and selenite, tellurite was removed from all cultures at a significantly (P < 0.05) higher percentage within 50 h. In a similar study, when *E. coli* was exposed to a mixture of oxyanions, the presence of selenite led to increased resistance to tellurite, which might be due to the fact that selenite can trigger higher oxidative stress than tellurite, thus producing a robust adaptive response, and preferential binding of selenite to tellurite target sites [57]. Bajaj and Winter also found that when more selenite was added into tellurite batches, faster tellurite reduction was observed, which is most likely due to the possibility that the presence of selenite activated additional enzymes such as glutathione reductase (an enhanced reductase activity) necessary to offset reactive oxygen species and tellurite [34]. However, [35] and [18] reported the opposite results.

The obtained results showed that using co-culture had more effect on selenite reduction than tellurite and improved the removal efficiency of selenite in separate cultures from 26.9 (*Y. lipolytica*) and 42.18 (*T. cutaneum*) to 56.17% in 50h. Meanwhile, efficiency of co-cultures in other batches was almost the same as pure cultures. In a research, a fungal-bacterial co-culture consisting of *Delftia* sp. and *Phanerochaete* sp. removed 10 mg/L (0.06 mM) of selenite while remediating 0.4 g/L of phenol [58]. It should be mentioned that a characteristic garlic-like odor was noticed in all the cultures, including tellurite, selenite, and tellurite/selenite cultures, which were even stronger in tellurite cultures, indicating the transformation of tellurite and selenite into the volatile organic Te and Se forms [59]. Overall, it can be suggested that the bioreduction of tellurite is more successful in the co-contaminant cultures associated with selenite than in the separate cultures within 50 h.

### The growth rate and kinetics of selenite and tellurite bioremoval by *Y. lipolytica* and *T. cutaneum*

**Fig. 2:**
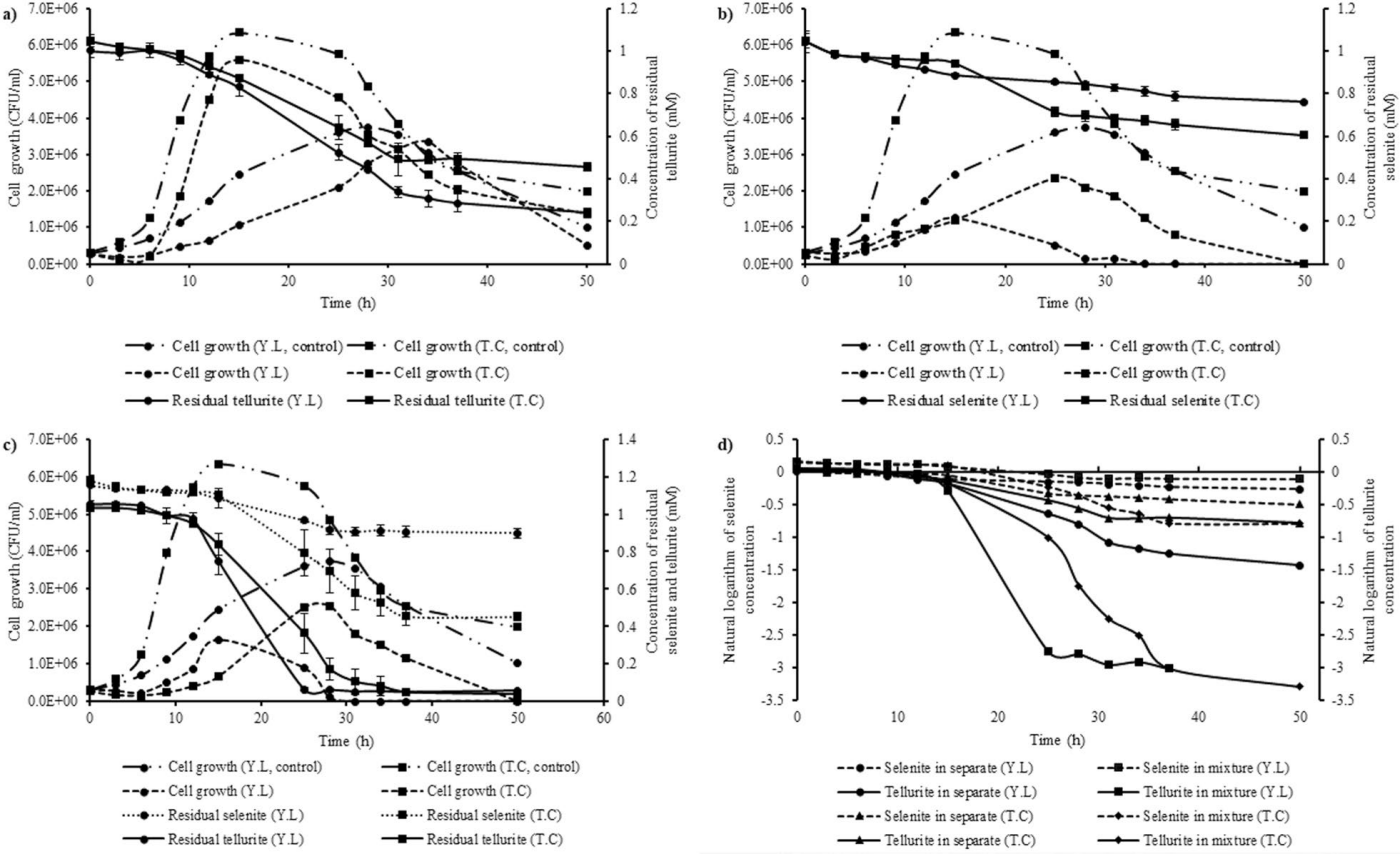
**a**–**c** The growth rate and reduction yield of *Y. lipolytica and T. cutaneum*, respectively, in tellurite (1 mM), selenite (1 mM), and tellurite (1 mM)/ selenite (1 mM) mixture; **d** the first-order removal model of tellurite and selenite in separate and mixture cultures. Values are mean ± SD of three replicates

In the bio-removal experiments, the kinetics of contaminant removal is recognized as an imperative and valuable method for forecasting, simplifying, and monitoring biological processes [60]. Figure 2 displays the growth curves and kinetics of tellurite and selenite removal. According to the figure, a direct connection can be noticed between the reduction of tellurite and selenite and growth. The reduction of tellurite and selenite displayed the same pattern, which shows that tellurite and selenite were removed at the same time.

During the reduction of tellurite in separate cultures, *Y. lipolytica* and *T. cutaneum* displayed three major growth phases, including lag, exponential, and death phases (Fig. 2a). Both strains did not show significant tellurite reduction during the lag period. The tellurite reduction rate reached its maximum during the exponential phase. While *T. cutaneum* had a shorter lag phase and an earlier and faster exponential phase than *Y. lipolytica*, their tellurite reduction followed the same pattern. And the number of living yeasts decreased in the death phase, and tellurite reduction was stopped.

The results of selenite reduction indicated that the same main growth phases as tellurite cultures’ growth were observed in separate cultures (Fig. 2b). Selenite reduction had not significantly occurred at the end of the lag phase. Most of the selenite reduction occurred during the exponential phase. Selenite was still reducing during the death phase at a slow rate, which shows that the reduction of selenite is accompanied by growth but is not confined to it. Cell growth and tellurite/selenite reduction in the co-contamination cultures followed the same patterns as the separate cultures.

The first-order model of the removal of tellurite and selenite is depicted in Fig. 2d, and Tables 2 and 3 show the correlation coefficient (R^2^), the removal rate constant (K), and half-life period (T_1/2_) of each used model of the two batches. Since the first-order model showed a higher R^2^ in all batches, tellurite and selenite reduction are consistent with the first-order model. The findings show that the K of tellurite reduction for *Y. lipolytica* and *T. cutaneum* were increased to 0.107 and 0.0811 per h, and the half-life decreased to 6.478 and 8.546 h, respectively, which shows tellurite reduction rate is faster in co-contaminant batches. In addition, the removal rate constant of selenite for *T. cutaneum* was increased to 0.0238 per h in the co-contamination cultures, and the half-life decreased to 29.123 h; however, in *Y. lipolytica* batches removal rate was almost the same as separate cultures.

**Table 2:** Kinetic parameters for 1 mM tellurite and 1 mM selenite removal in *Y. lipolytica* cultures

Parameters	Tellurite (separate)	Selenite (separate)	Tellurite (co-contamination)	Selenite (co-contamination)
Zero-order equation	Ct = − 0.0198 t + 1.064	Ct = − 0.0053 t + 0.9945	Ct = − 0.0352 t + 1.1994	Ct = − 0.0067 t + 1.1621
K_0_ (per h)	0.0198	0.0053	0.0352	0.0067
T_1/2_ (h)	26.868	93.82	17.037	86.72
R^2^	0.9324	0.9375	0.9157	0.8893
First-order equation	LnCt = − 0.0352 t + 0.171	LnCt = − 0.006 t −0.0025	LnCt = − 0.107 t + 0.6194	LnCt = − 0.0066 t + 0.154
K_1_ (per h)	0.0352	0.006	0.107	0.0066
T_1/2_ (h)	19.691	115.52	6.478	105.02
R^2^	0.9443	0.9558	0.9001	0.8904
Second-order equation	1/Ct = 0.0718 t + 0.5386	1/Ct = 0.0068 t + 0.9986	1/Ct = 0.6383 t − 2.896	1/Ct = 0.0065 t + 0.8532
K_2_ (per h)	0.0718	0.0068	0.6383	0.0065
T_1/2_ (h)	0.038	0.0068	1.848	0.0055
R^2^	0.9314	0.97	0.0901	0.8913

**Table 3:** Kinetic parameters for 1 mM tellurite and 1 mM selenite removal in *T. cutaneum* cultures

Parameters	Tellurite (separate)	Selenite (separate)	Tellurite (co-contamination)	Selenite (co-contamination)
Zero-order equation	Ct = − 0.0151 t + 1.065	Ct = − 0.0101 t + 1.0358	Ct = − 0.0273 t + 1.125	Ct = − 0.0185 t + 1.243
K_0_ (per h)	0.0151	0.0101	0.0273	0.0185
T_1/2_ (h)	35.264	51.277	20.604	30.891
R^2^	0.9223	0.9282	0.8978	0.919
First-order equation	LnCt = − 0.0211 t + 0.1011	LnCt = − 0.0126 t + 0.0506	LnCt = − 0.0811 t + 0.5236	LnCt = − 0.0238 t + 0.2779
K_1_ (per h)	0.0211	0.0126	0.0811	0.0238
T_1/2_ (h)	32.850	55.011	8.546	29.123
R^2^	0.9289	0.9395	0.9215	0.9153
Second-order equation	1/Ct = 0.031 t + 0.8351	1/Ct = 0.0101 t − 0.0358	1/Ct = 0.5019 t − 3.4761	1/Ct = 0.0326 t + 0.6543
K_2_ (per h)	0.031	0.0101	0.5019	0.0326
T_1/2_ (h)	0.025	0.00036	1.744	0.0213
R^2^	0.9297	0.9282	0.7998	0.8958

The maximum cell growth among all cultures was observed in the separate tellurite cultures. Additionally, the maximum growth for *Y. lipolytica* and *T. cutaneum* in co-contaminant cultures are 1.7E + 06 and 2.6E + 06 CFU/ml, respectively, which are 1.32 and 1.08 times higher than growth in separate selenite batches. Otherwise stated, the tellurite and selenite removal enhance as the growth increases. Although selenite cultures showed almost the same lag phases, tellurite batches of *Y. lipolytica* and *T. cutaneum* displayed different lag phases, which for *Y. lipolytica* achieved within about 12 h while for *T. cutaneum* were within 6 h of incubation. As shown in Fig. 2c, the lag phase of *T. cutaneum* in batches containing both contaminants ranged from 6 to 12 h, indicating the presence of a second toxic substance. The delay in the reduction and growth can be implied to the adaptability of the cells to tellurite and selenite.

### Characterization of biosynthesized Te–Se nanoparticles

**Fig. 3:**
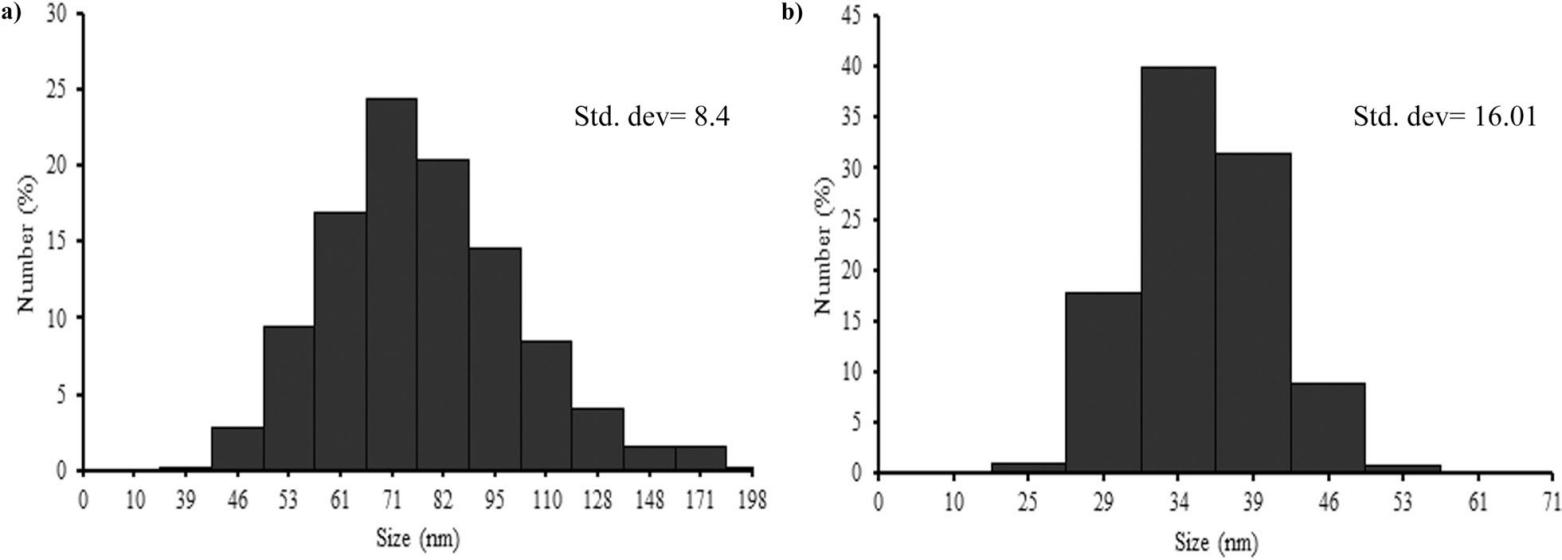
DLS analysis of *Y*. *lipolytica* (**a**) and *T.cutaneum* (**b**) of biosynthesized Te–Se NPs

The biosynthesis of Te–Se NPs has already been reported in a few studies by bacteria and fungi [18, 34], but as far as we know, the biosynthesis of Te–Se NPs by yeast species has not been reported yet. The DLS analyses displayed that the extracted Te–Se NPs of *Y. lipolytica* and *T. cutaneum* ranged from 46 to 171 nm and 25 to 53, respectively and the highest frequency was found in NPs with 71 and 34 nm (Fig. 3). In some similar studies, the sizes of Te–Se NPs generated by anaerobic granular sludge [6], *Phanerochaete chrysosporium* [18], *Duganella violacienigra*, and *Agrobacterium tumefaciens* [34] were ranging from 100–200, 50–600, 50–150, to 70–140 nm, respectively.

**Fig. 4:**
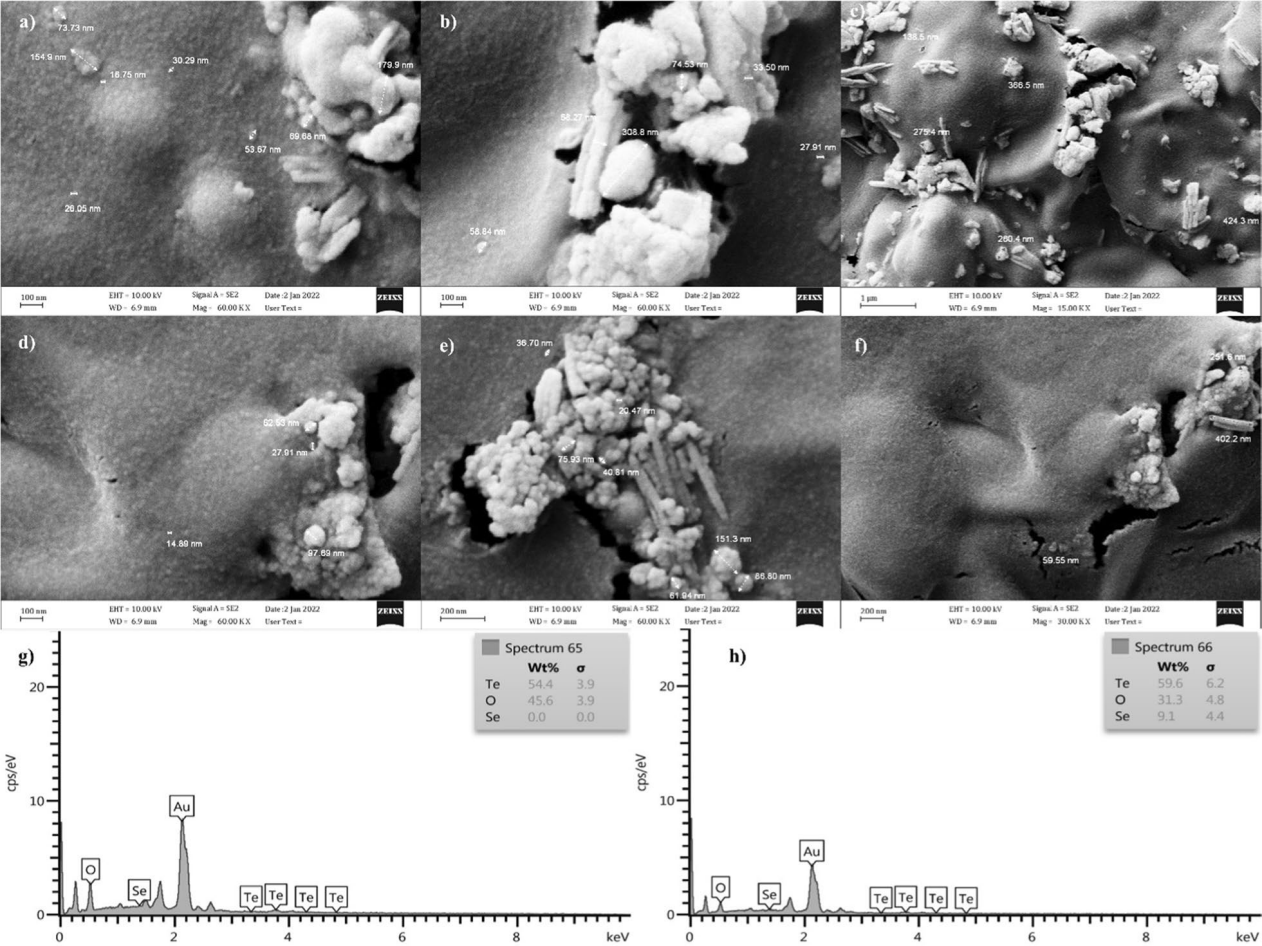
FE-SEM images of *Y.lipolytica* (**a**–**c**) and *T.cutaneum* (**d**–**f**) of the extracted Te–Se NPs, and EDX analyses of *Y. lipolytica* (**g**) and *T. cutaneum* (**h**)

The FE-SEM images (Fig. 4a–f) of Te–Se NPs showed that the nanoparticles synthesized by *Y. lipolytica* and *T. cutaneum* were both spherical and needle-like, which tended to aggregates to clusters of different sizes composed of smaller particles probably because of their high surface energy [61, 62]. Similar results were reported by [18], which stated that Te–Se NPs were spherical and needle-like. They also mentioned that the spherical NPs possessed a higher amount of Se, while needle-like NPs had a higher Te content. The size of nanoparticles has a significant influence on their application in several fields, such as catalysis, drug delivery, electronics, and imaging. Smaller NPs have a higher surface area to volume ratio (more active sites), can penetrate tissues and cells more easily (drug delivery and imaging), and offer improved conductivity (electronics) [63]. The NPs produced by these yeast strains were relatively small compared to similar studies, which shows their potential application in the mentioned fields.

**Fig. 5:**
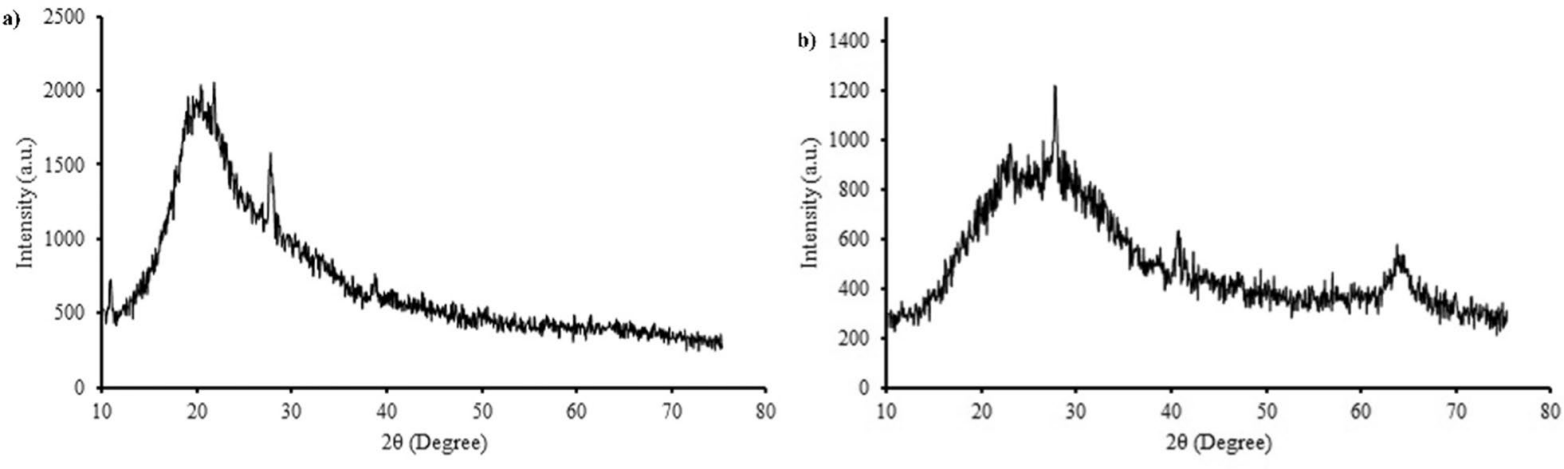
XRD patterns of *Y.lipolytica* (**a**) and *T.cutaneum* (**b**) of biosynthesized Te–Se NPs

According to EDX findings (Fig. 4g, h), which were consistent with the results of [6, 34], the elemental content of the Te–Se NPs was indicated to be elemental Te and Se. The XRD spectra of the Te–Se NPs showed no distinct peaks (Fig. 5), demonstrating an amorphous nature of the Te–Se NPs. Similar results were reported by [47] and [64], which showed that the produced Te and Se NPs in their studies were amorphous.

**Fig. 6:**
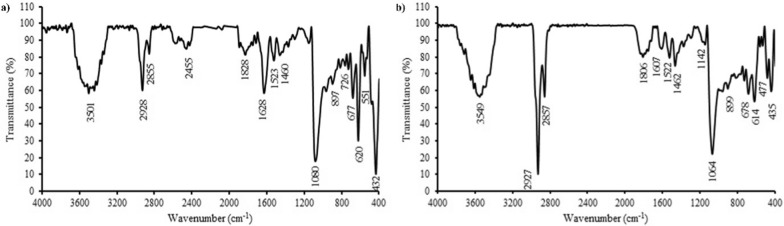
FTIR analyses of *Y.lipolytica* (**a**) and *T.cutaneum* (**b**) of biosynthesized Te–Se NPs

Figure 6 shows FTIR spectrums of Te–Se NPs biosynthesized by *Y. lipolytica* and *T. cutaneum*. FTIR analyses revealed several functional groups in Te–Se NPs, which shows the nanoparticle surface was capped by lipids, proteins, and carbohydrates [64, 65]. Both spectrums showed a broad band at 3500 cm^−1^, which corresponds to O–H or the N–H stretching vibrations of amide A in proteins [65, 66]. Also, the peak at 2900 cm^−1^ in both strains can be attributed to the C-H stretching of the methylene groups of lipids [64, 65]. The peak at 2455 cm^−1^ in the *Y. lipolytica* spectrum confirmed the S–H stretching of thiols [66]. The absorption bands at 1800 cm^−1^ in both spectrums can indicate the C = O stretching related to esters [65]. The peaks at 1500–1600 cm^−1^ in both spectrums can be attributed to the N–H bending of primary and secondary amine in proteins [66]. The peaks at 1460 cm^−1^ in both strains correspond to C–H bending in methylene [66], and also the sharp peak at 1070 cm^−1^ can be attributed to stretching C–C/C–O in polysaccharides or similar functional groups in proteins and polyesters [65]. The peaks at 700–900 cm^−1^ and 400–600 cm^−1^ can correspond to stretching C–Cl/C–F in aliphatic compounds, and to stretching S–S in polysulfides, aryl disulfides, and disulfides, respectively.

Similar signals between the FTIR spectra can be due to the existence of common pathways for the synthesis of these nanoparticles in both yeasts. Although, differences in the intensity of some signals are observed in their spectra. For example, in 2927 cm-1, a more intense signal is observed for *T. cutaneum* compared to *Y. lipolytica* nanoparticles, which can be due to the presence of some long chain linear aliphatic compounds in the coating of these nanoparticles. Also, the ratio of CH2/CH3 is higher for *T. cutaneum*. In addition, the signal of 1628 cm^-1^ is present only in the nanoparticles obtained from *Y. lipolytica*, which corresponds to the amide I and II. Also, the signal of 620 cm^-1^ has a higher intensity in the spectrum of nanoparticles obtained from *Y. lipolytica*, which is related to polysulfide, aryl sulfide, and disulfide. It is concluded that nanoparticles produced by *Y. lipolytica* have protein in their cap layer compared to those of *T. cutaneum* that contained lipids [66].

The presence of thiol in the FTIR spectrum of *Y. lipolytica* may refer to the mechanism of nanoparticle formation. The production of selenium and tellurium nanoparticles occurred by various mechanisms, among which reductase enzymes, such as glutathione reductase and thioredoxin reductase can reduce these oxyanions by electrons donated by thiol-containing proteins and peptides [67], investigated the role of these enzymes in the reduction of selenite and tellurite in yeast. In addition to the thioredoxin system and thiol-containing proteins such as glutathione, other mechanisms involved in the reduction of these two oxyanions in *Bacillus mycoides* SeITE01 have been investigated [68]. Also, [69] and [70] suggested that thiol-containing peptides play a critical role in biosynthesis and stabilization of nanoparticles in *Stenotrophomonas bentonitica*.

## Conclusion

This study reports the use of yeast strains and their co-culture in the co-contaminant bioreduction of tellurite and selenite, associated with biosynthesis and characterization of Te–Se NPs for the first time, which indicates the great potential of yeast cultures for reduction of contaminated sites and Te–Se NPs production. Tellurite and selenite both showed inhibitory behavior, and the first-order kinetics model could accurately predict their removal kinetics. In separate cultures, *Y. lipolytica* batches performed better in removing tellurite and showed the lowest selenite reduction efficiency. In addition, *Y. lipolytica* and *T. cutaneum* in co-contaminant cultures displayed the highest tellurite reduction efficiencies. Besides, the efficiency of co-cultures was almost the same as pure cultures. Overall, co-contaminant cultures showed higher efficiency in the bioreduction of metalloid oxyanions than separate cultures. FTIR, DLS, EDX, XRD, and FE-SEM data showed that the amorphous Te–Se NPs were covered with lipids, carbohydrates, and proteins and ranged from 25 to 171 nm in size. This method suggests a cost-effective, green, and eco-friendly method for the simultaneous removal of tellurite and selenite in polluted sites and Te–Se NPs synthesis for nanotechnology, medicine, and industrial applications.

### Supplementary Information

** Additional file 1: Table S1.** Evaluation of tellurite and selenite reduction capabilities of different yeast strains based on color intensity after 48 h. **Figure S1.** Bioreduction of 1 mM selenite (**a**), 1 mM tellurite (**b**), and their mixture (**c**) by *Y.lipolytica*, *T.cutaneum*, and their co-cultures after 48 h.

## Data Availability

The data that support the findings of this study are available from the corresponding author upon reasonable request.
