# Supplementary material for: Simultaneous bioreduction of tellurite and selenite by Yarrowia lipolytica, Trichosporon cutaneum, and their co-culture along with characterization of biosynthesized Te–Se nanoparticles
Source: Microb Cell Fact. 2023 Sep 25;22:193. doi: 10.1186/s12934-023-02204-0 (PMC10519092; doi:10.1186/s12934-023-02204-0)
Supplement: Supplementary file 1 — Additional file 1: Table S1. Evaluation of tellurite and selenite reduction capabilities of different yeast strains based on color intensity after 48 h. Figure S1. Bioreduction of 1 mM selenite (a), 1 mM tellurite (b), and their mixture (c) by Y.lipolytica, T.cutaneum, and their co-cultures after 48 h. [file 12934_2023_2204_MOESM1_ESM.pdf]

Simultaneous bioreduction of tellurate and selenite by Yarrowia lipolytica, Trichosporon cutaneum, and their co-culture along with characterization of biosynthesized Te-Se nanoparticles

Running Head: Co-remediation of two metalloid oxyanions by yeast strains

Firooz Hosseini, Maryam Hadian, Elham Lashani, Hamid Moghimi*

Department of Microbial Biotechnology, School of Biology, College of Science, University of Tehran, Tehran, Iran

Corresponding author: Hamid Moghimi

Department of Microbial Biotechnology, School of Biology, College of Science, University of Tehran, Tehran, Iran.

Tel: +98-21-66113314

Fax: +98-21-66415495

Postal code: 1417864411

Email: hmoghimi@ut.ac.ir

Table S1
Evaluation of tellurite and selenite reduction capabilities of different yeast strains based on color intensity after 48 h.

Strain	Tellurite reduction	Selenite reduction	Mixture
Y.lipolytica	+++	+++	+++
T.cutaneum	+++	+++	+++
10	+++	+++	++
8	+++	-	++
5	+++	+	+++
S. pombe	+++	++	-
126	+++	++	+++
123	+++	-	++
S. cerevisiae	++	++	+
25	+	+++	-
129	-	+	+
66	-	+	-

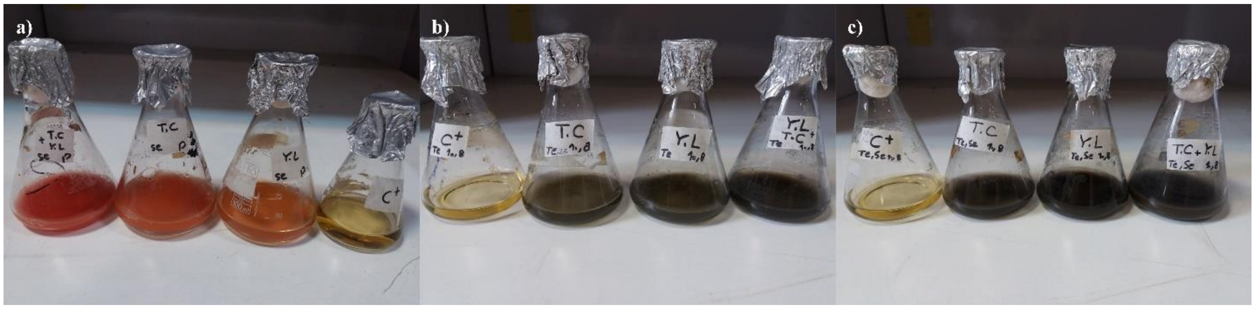

Fig.S1. Bioreduction of 1mM selenite (a), 1mM tellurite (b), and their mixture (c) by *Y.lipolytica*, *T.cutaneum*, and their co-cultures after 48 h.
